# Video display terminal use and other risk factors for abnormal blinking in children: gender differences

**DOI:** 10.1186/s12886-021-02194-w

**Published:** 2021-12-10

**Authors:** Hui Zhao, Shi-Nan Wu, Qi Zhang, Chen Zhao, Hui-Ye Shu, Qian-Min Ge, Yi Shao

**Affiliations:** 1 Xinhua Hospital Affiliated To Shanghai Jiao Tong University School of Medicine, Shanghai, 200092 China; 2 Department of Radiology, Jiangxi Province Medical Imaging Research Institute, The First Affiliated Hospital of Nanchang University, Jiangxi 330006 Nanchang, People’s Republic of China; 3 Department of Ophthalmology, Jiangxi Province Ocular Disease Clinical Research Center, The First Affiliated Hospital of Nanchang University, No 17, YongWaiZheng Street, DongHu District, Jiangxi 330006 Nanchang, China

**Keywords:** Children, Abnormal blinking, Video display terminals

## Abstract

**Objective:**

To explore the risk factors for abnormal blinking in children and compare these between boys and girls.

**Methods:**

Children attending the Children's Optometry Clinic between June 2019 and June 2020 were recruited for the study. The time they had spent viewing video displays (VDTt) over the past 6 months was recorded. Incomplete blinking (IB) and blinking rate were measured and all participants were allocated to groups based on their blink rate (<20 times/min = normal blinking group, NBG; ≥20 times/min = abnormal blinking group, ABG). Tear film (TF) stability was also evaluated. The corresponding statistical methods are used to analyze the data.

**Results:**

A total of 87 boys and 80 girls were enrolled in the study. No significant difference in age was found between the 2 groups. There was a significant difference in TF stability between the two groups (*P*<0.05). According to binary logistic analysis, VDTt and ocular protection index (OPI) are important risk factors for abnormal blinking, with cut-off values of 1.75 hours and 1.014 respectively in boys; and 1.25 hours and 1.770 respectively in girls. The average of lipid layer thickness was an important protective factor for children using VDT for long periods, with a cut-off value of 58.5 nm in boys and 53.5nm in girls.

**Conclusion:**

Risk factors for abnormal blinking in both boys and girls include VDTt and OPI.

## Introduction

Abnormal blinking in children is a symptom frequently encountered by ophthalmologists in the outpatient clinic. Relevant studies have confirmed that the most common causes of excessive blinks in children are eyelid abnormalities, habitual eye muscle convulsions, uncorrected refractive errors, intermittent exotropia, cardiogenic blepharospasm, etc [1]. However, the relationship between the use of terminal devices and abnormal blinking in children’s daily video exposure has not been studied. Some ophthalmologists respond to this symptom with eye drops for conjunctivitis, but in fact, abnormal blinking is often one of the main symptoms of dry eyes [2]. Tears are produced by the lacrimal glands, distributed by blinks, evaporated from the ocular surface, and drained through the nasolacrimal ducts. Any abnormalities in the above steps may result in dry eyes [3]. For example, in some situations and diseases, tear evaporation may increase. In the case of video display terminal (VDT) operation, the evaporation of tears increases due to the decrease in blink frequency. With the diversification of VDT technology, children use VDTs at earlier ages and for longer periods. Prolonged exposure to VDT has been shown to be associated with myopia and dry eyes [4, 5]. Moreover, in the ophthalmology clinic, the number of children complaining of abnormal blinking is rapidly increasing. At present, the specific etiology of abnormal blinking in children is still relatively unknown. To date, studies have confirmed a strong correlation between abnormal blinking and dry eyes, which can be exacerbated by staring and blinking hard while reading for long periods [6].

The meibomian glands are located in the tarsal palpebral plate. The glands are perpendicular to the margin of the eyelid, arranged in parallel, with openings in front of the posterior lip of the eyelid margin, where they secrete lipids. In the process of blinking, the lipids are distributed on the tear film (TF) surface, forming a lipid layer [7]. The lipid layer can prevent the rapid evaporation of tears, improve the stability of the TF, provide a smooth optical interface, reduce the damage caused by blinks, prevent the TF from being polluted by sebaceous gland secretions, lubricate the eyelid and eye surface, and prevent tear loss [8].

Since some studies have shown that abnormal blinking is nearly twice as common in boys as girl s[1]. The differences may be due to environmental factors and hormonal levels. Sex hormones are involved in the physiology and pathophysiology of almost all organs of the human body and most diseases [9]. Recently, several studies have focused on the relationship between sex hormones and ocular surface tissue. Androgens have been shown to have a positive effect on dry eyes, while the effect of the corresponding estrogen on eye disease is unclear [10]. In a study of older men, positive associations were found between levels of androstenediol and physical health, lipids, and water-based TF parameters [11]. Research has also confirmed that smartphone use is a significant risk factor for dry eye disease in children, and prolonged use may increase this risk [4]. However, the study did not explore specific differences in risk factors between boys and girls. Therefore, the present study investigated the risk factors for abnormal blinking in boys and girls respectively and compared them according to their specific critical thresholds.

## Methods

### Patients and examination

The study included 87 boys and 80 girls attending the ophthalmology clinic of Xinhua Hospital Affiliated to Medical College of Shanghai Jiao Tong University between June 2019 and June 2020. All participants were allocated to groups based on their blink rate: ≥20 blinks per minute = abnormally blinking group (ABG); <20 blinks per minute = normal blinking group (NBG) [12].

Children meeting the following criteria were eligible to participate: (1) no active eye inflammation and no use of eye drops within the preceding three months; (2) no history of wearing corneal contact lenses, laser or other ocular surgery in the preceding six months, no history of ocular trauma, ocular chemical injury or burns; (3) no upper respiratory tract infection within the preceding two weeks; (4) no use of atropine, neostigmine, artificial tears and other drugs affecting tears within the preceding six months.

**Fig. 1:**
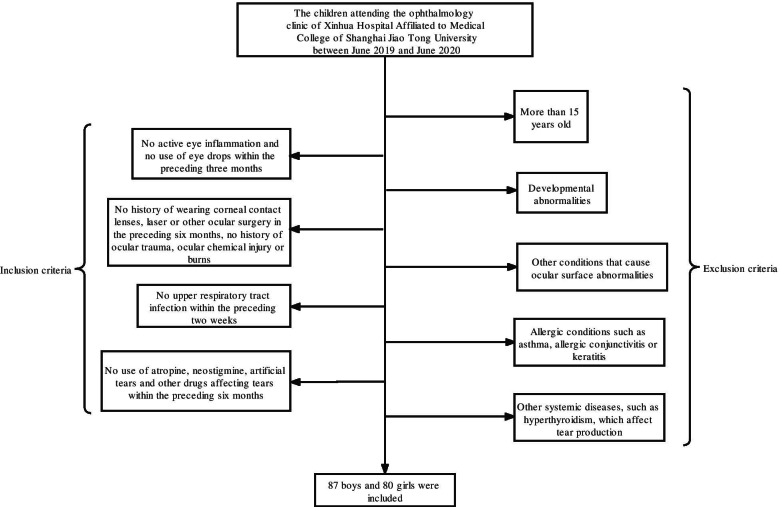
Flow chart of inclusion and exclusion criteria for subjects

Children were not eligible if they were older than 15 years or had one of the following conditions: (1) other conditions that cause ocular surface abnormalities, such as abnormal eyelid position, ocular prolapse, or pterygium; (2) allergic conditions such as asthma, allergic conjunctivitis or keratitis; (3) other systemic diseases, such as hyperthyroidism, which affect tear production; or (4) developmental abnormalities (Fig. 1). The study was consistent with the principles of the Declaration of Helsinki. The child's guardian signed a declaration of informed consent. The study was approved by the Ethics Committee of Xinhua Hospital and was registered with the Chinese Clinical Trial Registry (Trial Registration No: ChiCTR2000038908).

Eye examinations and other data were collected by one ophthalmologist in the following order: (1) the child’s usage time of VDT (VDTt) was collected through a questionnaire which was completed by the child and/or guardian; (2) Lipiview® (Tear Science, Morrisville, NC, USA) measurements (including lipid layer thickness (LLT) and blink rate) were recorded [13]; (3) tear meniscus height (TH) was measured and meibomian gland (MG) morphology was assessed using the Oculus Keratography 5M (Oculus, Wetzlar, Germany) [14, 15]; (4) an ocular surface slit lamp examination was carried out to measure conjunctival congestion assessment and fluorescein tear film breakup time (BUT), the Marx line (ML) score, Meibomian gland expression and Meibum score, and corneal fluorescein staining score.

### Measurement of LLT, incomplete blinks (IB) and blink rate

The LLT of the TF (minimum (MIN), maximum (MAX) and average (AVG)), number of IB and the blink rate over a 10 s period were measured using a Lipiview® interferometer and during this procedure the child was encouraged to blink naturally.

### TH and MG morphology assessment

#### Slit lamp examination

Conjunctival congestion was assessed using the Cornea and Contact Lens Research Unit (CCLRU) classification standard as the reference point, with a generic (0-4) scale to score redness and roughness of the palpebral conjunctiva [16].

To measure BUT, 2 μL 1% sodium fluorescein preservative free solution was dropped into the lower conjunctival sac; patients were instructed to blink their eyes several times for a few seconds, measurement was made three times, and the mean BUT was calculated. In addition, the ocular protection index (OPI) was calculated using the ratio of BUT to blink interval (blink interval = 60 / blink rate). The OPI has previously been used to assess factors that may cause or exacerbate dry eye syndrome [17].

The corneal fluorescence staining score (FL) was measured by considering the corneal surface as four quadrants: supra-nasal, infra-nasal, superior temporal, and inferior temporal, with each quadrant being scored I-IV for a total out of 16 points. Scoring criteria were: I (no staining); II (mild scattered spot staining, 1-30 spots stained); III (moderate staining, >30 spots stained but staining not fused); IV (heavy staining or sheet staining of the entire cornea, spot staining fused, with filaments or ulcers) [18].

The Marx line (ML) score was calculated for the outer, middle, and inner thirds of the lower eyelid margin, and recorded as: I (entirely on the conjunctival side of the meibomian orifices (MOs)); II (part of the ML touches the MOs); III (ML runs through all of the MOs); and IV (ML runs on the eyelid margin side of the MOs) [19–21].

To measure MG expression (MGE), the five glands at the center of the upper and lower eyelids were located and their orifices observed. Scores were based on the following scale: I (normal, meibum secretion from all glands with light pressure on the eyelid); II (secretion from 3 to 4 glands with light pressure on the eyelid; III (secretion from 1 to 2 glands with light pressure on the eyelid); IV: no secretion from any glands with light pressure on the eyelid) [22].

The quality of secreted meibum was scored (Meibum score) according to the following criteria: I (normal, clear, transparent lid ester); II (cloudy lid ester); III (cloudy lid ester with granules); IV (thick lid fat with a toothpaste-like consistency) [23].

#### Statistical analyses

Statistical analysis was conducted using SPSS version 25.0 (USA, Chicago, Illinois, SPSS) and R version 4.0.5 (packages including plyr version 1.8.6, readxl version 1.3.1, DescrTab2 version 2.0.7, pROC version 1.18.0, ggplot2 version 3.3.5, foreign version 0.8-81 and rms version 6.2-0) software to carry out t-tests, Chi-square test and Fisher’s Exact Test between groups and to draw the ROC Curve. Binary logistic analysis was performed on the related factors according to the grouping. Receiver operating characteristic (ROC) curves were used to analyze the significance of different groups for differential diagnosis of risk factors, and the area under the curve (AUC) corresponding to different indicators and the critical threshold for differential diagnosis were calculated. *P* values of less than 0.05 were considered statistically significant.

## Results

### Patients demographics

A total of 167 right eyes were examined (87 boys). The mean age of boys in NBG was 7.49 +/- 2.95 (2-15 years old, 43 children), and in ABG was 7.93 +/- 2.61 (2-15 years old, 44 children) with no significant difference (*P*=0.460; Table 1). The mean age of girls in NBG was 8.18 +/- 2.27 (2-15 years old, 40 children) and in ABG was 8.38 +/- 3.41 (2-15 years old, 40 children) with no significant difference (*P*=0.759; Table 1).

### Blink rate

In both boys and girls, blinking rate, incomplete blinking rate and VDTt were all significantly higher in ABG than NBG (P<0.001).

### The stability of tear film

For evaluation and analysis of TF stability, TH, BUT, and average (AVG), minimum (MIN), maximum (MAX) indices of the thickness of TF lipid layer as well as OPI values were compared between groups and genders. In boys, BUT, TH, AVG, MIN and MAX were significantly higher in NBG than in ABG (*P*<0.003), OPI was significantly higher in ABG than NBG (*P*=0.004) and no significant difference was found in FL. Similarly, AVG, MAX and MIN were significantly higher in girls in the NBG than ABG groups, and no significant difference in FL was found between the two groups, However, in contrast with data from boys, BUT and TH were statistically similar in girls in the NBG and ABG groups (Fig. 1 and Table 1).

**Table 1:** General information and the index of tear film

Gender	Parameters	NBG	ABG	*X*^*2*c^	P value^c^
Male	Age (years)^a^	7.49±2.95	7.93±2.61		0.460
	Blinking rate^a^	14.65±3.77	31.5±8.69		<0.001
	Incomplete blinking rate^a^	13.26±8.02	23.86±12.58		<0.001
	VDTt (hours)^a^	1.04±0.88	2.69±0.91		<0.001
	FL^b^			1.347	1.000
	I	42(98%)	41(93%)		
	II	0(0%)	1(2%)		
	III	1(2%)	2(5%)		
	BUT(s) ^a^	6.43±2.68	4.29±2.43	-	<0.001
	TH (mm) ^a^	0.23±0.07	0.18±0.07	-	0.002
	AVG^a^	69.70±22.43	43.34±13.22	-	<0.001
	MIN^a^	61.40±22.77	38.66±12.85	-	<0.001
	MAX^a^	80.35±21.33	54.45±15.14	-	<0.001
	OPI^a^	1.56±0.76	2.19±1.21	-	0.004
Female	Age (years)^a^	8.18±2.27	8.38±3.41		0.759
	Blinking rate^a^	14.55±4.27	28.35±5.92		<0.001
	Incomplete blinking rate^a^	14.70±6.22	23.55±10.20		<0.001
	VDTt (hours) ^a^	1.01±1.03	2.24±1.21		<0.001
	FL^b^			0.732	0.753
	I	35(87.5%)	37(92.5%)		
	II	3(7.5%)	2(5%)		
	III	0(0%)	0(0%)		
	IV	2(5.0%)	1(2.5%)		
	BUT(s) ^a^	6.06±2.60	5.34±2.80	-	0.236
	TH (mm) ^a^	0.20±0.08	0.19±0.07	-	0.815
	AVG^a^	71.78±21.87	52.50±19.20	-	<0.001
	MIN^a^	66.40±21.84	46.90±18.82	-	<0.001
	MAX^a^	82.23±21.43	64.73±23.62	-	0.001
	OPI^a^	1.51±0.86	2.52±1.52	-	0.001

Notes: Data showed as mean +/- standard deviation or n

Abbreviation: NBG, normal blinking group; ABG, abnormal blinking group; VDTt, video display terminal time. FL, corneal fluorescence staining score; BUT, tear film breakup time; TH, tear meniscus height; AVG, the average of tear film lipid layer thickness; MIN, the minimum of tear film lipid layer thickness; MAX, the maximum of tear film lipid layer thickness; OPI, ocular protection index

*P* < 0.05 indicates statistical significance

^a^ Independent sample T test

^b^ Pearson Chi-Square and Fisher’s Exact Test

^c^ Comparison between the NBG and ABG

### Evaluation of Meibomian gland function

**Fig. 2:**
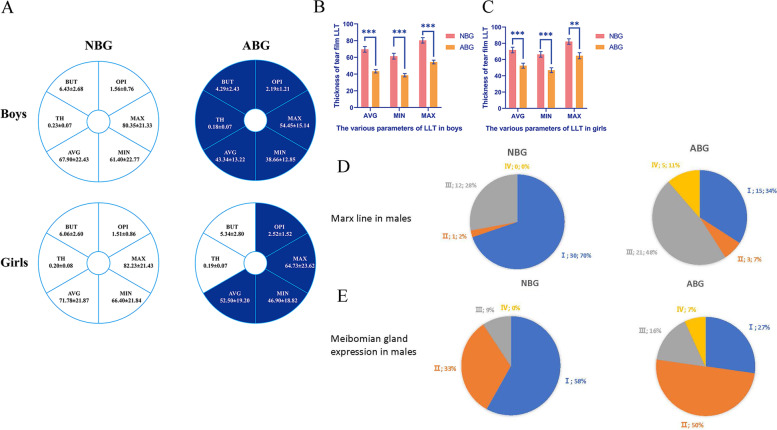
Comparison of NBG and ABG on FL indicators, index of tear film and meibomian gland function in boys and girls. *** There was a significant difference between the two groups; *P* value <0.001. ** There was a significant difference between the two groups; *P* value <0.01. Notes: **A **showed the difference of index of tear film; **B** showed the difference of tear film LLT between NBG and ABG in boys; **C** showed the difference of tear film LLT between NBG and ABG in girls. In the case of gender, horizontal comparison of indicators with color difference represents significant difference. **D** showed the comparison of marx line between NBG and ABG in boys; **E** showed the comparison of meibomian gland expression between NBG and ABG in boys. Abbreviation: LLT, lipid layer thickness; NBG, normal blinking group; ABG, abnormal blinking group; BUT, tear film breakup time; TH, tear meniscus height; AVG, the average of tear film lipid layer thickness; MIN, the minimum of tear film lipid layer thickness; MAX, the maximum of tear film lipid layer thickness; OPI, ocular protection index.

LLT is closely related to MG function [24]. In our study, MG dropout, Meibum score, MGE and ML were used to evaluate the function and morphology of MG. MG dropout and Meibum scores were not statistically significant between boys in NBG and ABG or girls in NBG and ABG (*P*>0.05). However, there were significant differences in ML and MGE between the two group in boys (*P*<0.05). More information is shown in Table 2 and Fig. 2.

**Table 2:** The function of meibomian gland

Gender	Parameters	NBG	ABG	*X*^2^**	*P* value**
Male	ML^a^			13.445	0.002
	I	30(70%)	15(34%)		
	II	1(2%)	3(7%)		
	III	12(28%)	21(48%)		
	IV	0(0%)	5(11%)		
	MGE^a^			10.153	0.012
	I	25(58%)	12(27%)		
	II	14(33%)	22(50%)		
	III	4(9%)	7(16%)		
	IV	0(0%)	3(7%)		
	MG dropout^a^			1.035	0.494
	I	42(98%)	44(100%)		
	II	1(2%)	0(0%)		
	Mebium score^a^			2.388	0.314
	I	41(95%)	38(86%)		
	II	2(5%)	4(9%)		
	III	0(0%)	2(5%)		
Female	ML^a^			4.780	0.180
	I	29(72.5%)	21(52.5%)		
	II	2(5%)	2(5%)		
	III	9(22.5%)	15(37.5%)		
	IV	0(0%)	2(5%)		
	MGE^a^			5.513	0.107
	I	26(65%)	16(40%)		
	II	8(20%)	12(30%)		
	III	6(15%)	11(27.5%)		
	IV	0(0%)	1(2.5%)		
	MG dropout^a^			2.817	1.000
	I	39(97.5%)	38(95%)		
	II	0(0%)	1(2.5%)		
	III	1(2.5%)	0(0%)		
	IV	0(0%)	1(2.5%)		
	Mebium score^a^			4.342	0.171
	I	39(97.5%)	34(85%)		
	II	0(0%)	3(7.5%)		
	III	1(2.5%)	3(7.5%)		

*P*<0.05 indicates statistical significance

^a^Pearson Chi-Square and Fisher’s Exact Test

**Comparison between the NBG and ABG

*Abbreviations:NBG *normal blinking group, *ABG *abnormal blinking group, *ML *marx line, *MGE *meibomian gland expression, *MG* dropout, meibomian gland dropout degree

### Binary logistic regression and ROC curve analysis

Binary logistic multiple analysis on data from both genders in the ABG and NBG groups showed that VDTt and OPI were important risk factors. In boys, the odds ratios (OR) values of the two indices were 10.427 (95% CI: 3.897-27.895) and 5.267(95% CI: 1.885-14.720), respectively. Based on these two risk factors, ROC curves were used for differential diagnosis analysis. The AUC value corresponding to VDTt was 0.885, while the value corresponding to OPI was 0.664. The sensitivity, specificity and cut-off values of the two indicators were 0.932, 0.814 and 1.75 (*P*<0.001); 0.909, 0.372 and 1.014 (*P*=0.008). In girls, VDTt and OPI were similarly important risk factors for abnormal blinking, and their OR values were 2.762 (95% CI: 1.618-4.712) and 2.602 (95% CI: 1.375-4.921), respectively. ROC curve analysis showed that the AUC values of VDTt and OPI were 0.783 and 0.704 respectively. The sensitivity, specificity, and cut-off values of the two indices were calculated as 0.725, 0.850, and 1.25 (*P*<0.001); 0.675,0.675, and 1.770 (*P*=0.002).

### Binary logistic regression and ROC curve analysis based on cut-off values of VDTt

Data from boys were grouped according to the 1.75 hours cut off value calculated previously, and binary logistic multiple analysis was performed. This showed that AVG is an important protective factor with OR value of 0.797 (95% CI: 0.708-0.897). ROC curve analysis showed that the AUC value of AVG index was 0.962, and the sensitivity, specificity and cut-off values were 0.816, 0.980 and 58.5, respectively.

**Fig. 3:**
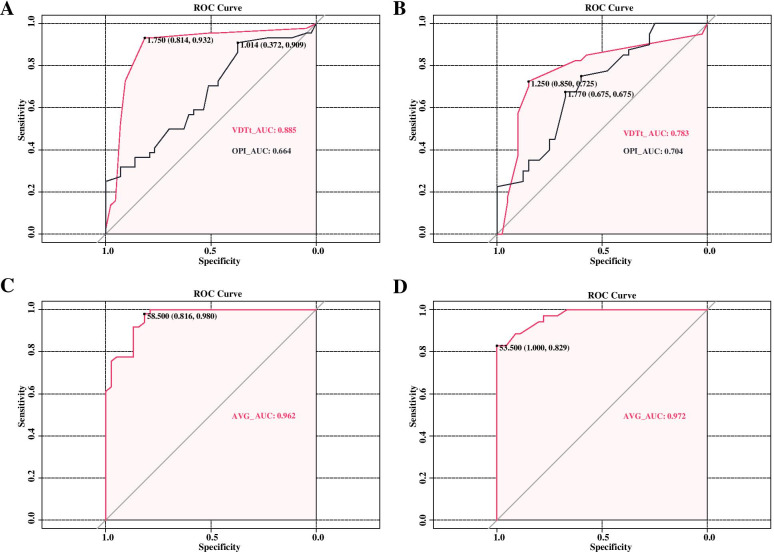
**A** and **B**. The receiver operating characteristics (ROC) curves of risk factors for detecting children with abnormal blinking. Notes: **A**: ROC curves of VDTt and OPI showed that the AUC values were 0.885 and 0.664, respectively (*P*<0.001, *P*=0.008). The sensitivity and specificity were 93.2% and 81.4%; 90.9% and 37.2%, respectively in boys. **B**: ROC curves of VDTt and OPI showed that the AUC values were 0.783 and 0.704, respectively (*P*<0.001, *P*=0.002). The sensitivity and specificity were 72.5% and 85.0%; 67.5% and 67.5%, respectively in girls. Abbreviations: VDTt, video display terminal time; OPI, ocular protection index; AUC, area under the curve (**C**) and (**D**). The receiver operating characteristics (ROC) curves for factors associated with prolonged use of VDT in children. Notes: **C**: ROC curves of AVG showed that the AUC value was 0.962 (*P*<0.001). The sensitivity and specificity were 81.6% and 98.0%, respectively in boys. **D**: ROC curves of AVG showed that the AUC value was 0.972 (*P*<0.001). The sensitivity and specificity were 100% and 82.9%, respectively in girls. Abbreviation: AVG, the average of tear film lipid layer thickness; AUC, area under the curve

Girls were grouped according to the 1.25 hour cut off calculated previously. Binary logistic multiple analysis again showed that AVG was an important protective factor, and its OR value was 0.672 (95% CI: 0.523-0.864). The subsequent ROC curve analysis found AUC value of AVG to be 0.972, and its sensitivity, specificity and cut-off values were 1.000, 0.829 and 53.5 respectively. More detailed data are shown in Tables 3 and 4, and Fig. 3.

**Table 3:** Binary logistic analysis results under different groups.

Gender	Factors	B	OR	OR (95% CI)	*P* Value
Male	VDTt^a^	2.344	10.427	3.897-27.895	<0.001
	OPI^a^	1.661	5.267	1.885-14.720	0.002
	AVG^b^	-0.227	0.797	0.708-0.897	<0.001
Female	VDTt^a^	1.016	2.762	1.618-4.712	<0.001
	OPI^a^	0.956	2.602	1.375-4.921	0.003
	AVG^c^	-0.397	0.672	0.523-0.864	0.002

^a^The results are grouped according to a bound of 20 blinks per minute

^b^The results are grouped according to a daily bound value of 1.75 hours of VDT usage

^c^The results are grouped according to a daily bound value of 1.25 hours of VDT usage

*Notes: P*<0.05 denoted statistical significance

*Abbreviations:B *coefficient of regression, *OR *odds ratio, *CI *confidence interval, *VDTt *video display terminal time, *OPI *ocular protection index, *AVG *the average of tear film lipid layer thickness

**Table 4:** The Cut-off Value, sensitivity, specificity, and AUC of factors for the different grouping conditions

Gender	Factor	Cut-Off Value	Sensitivity (%)	Specificity (%)	AUC	*P* Value
Male	VDTt^a^	1.750	0.932	0.814	0.885	<0.001
	OPI^a^	1.014	0.909	0.372	0.664	0.008
	AVG^b^	58.500	0.816	0.980	0.962	<0.001
Female	VDTt^a^	1.250	0.725	0.850	0.783	<0.001
	OPI^a^	1.770	0.675	0.675	0.704	0.002
	AVG^c^	53.500	1.000	0.829	0.972	<0.001

^a^The results are grouped according to a bound of 20 blinks per minute

^b^The results are grouped according to a daily bound value of 1.75 hours of VDT usage

^c^The results are grouped according to a daily bound value of 1.25 hours of VDT usage

*Notes*: *P*<0.05 denoted statistical significance

*Abbreviations*: *VDTt *video display terminal time, *OPI *ocular protection index, *VG *the average of tear film lipid layer thickness, *AUC *area under the curve

## Discussion

In our study, VDTt and OPI were shown to be important risk factors for abnormal blinking in children. The indexes of tear film lipid layer thickness in abnormal blinking group were significantly lower than those in normal blinking group. In boys, the expression levels of Marx line and meibomian gland in normal blink group were significantly higher than those in abnormal blink group. Long-term use of VDT for more than 1.75 hours per day in boys and 1.25 hours per day in girls is a concern for abnormal blinking. The critical thresholds for OPI were 1.014 for boys and 1.770 for girls. In addition, we found that mean TF lipid layer thickness was an important protective factor for long-term use of VDT in both boys and girls, with critical thresholds of 58.5 nm and 53.5 nm respectively. These results suggest that girls should spend slightly less time using VDT than boys to better protect ocular surface health. In terms of long-term use of VDT, the thickness of the TF lipid layer corresponding to girls has a wider effective protective range.

To date, studies have shown that excessive blinking in children is rarely an indication of a neurological disorder and usually resolves over time without intervention [25]. However, in clinical practice, the number of children with abnormal blinking as the main complaint is increasing, and the clinical symptoms are often reported as frequent or forced blinking. Blinking is one of the most frequent movements in healthy subjects. This action involves the interaction of at least two muscles: transient phased activation of the orbicularis oculi muscle and transient inhibitory activity of the levator palpebrae muscle surface [26]. Studies have shown that abnormal blinking and dry eyes are important risk factors for lid wiper epitheliopathy (LWE) [27]. Therefore, the effective evaluation and analysis of abnormal blinking has important application in clinical practice. In our study, VDTt and OPI are important risk factors for abnormal blinking in children.

A considerable number of studies have confirmed that VDT is closely related to various eye disorders. These include myopia, dry eyes and blurred vision [28, 29]. In China, one of the prime risk factors for myopia in children is prolonged close smartphone use [30]. In the case of dry eye, studies have shown that gender and hormones play an important role in the regulation of ocular surface, adnexal tissue, and the gender difference in prevalence of dry eye [31]. Epidemiological studies have found that the prevalence of dry eye is 33% in the Asian population, and that increasing age, female gender, smoking, use of video display devices and environmental stress are all important factors contributing to the exacerbation of the disease [32]. In addition, the lipid layer of the TF may be thinner and more contaminated in women than men of similar ag e[33] providing further evidence that women are at significantly higher risk of developing dry eyes than men. Moreover, studies have confirmed that corneal epithelium and stroma thicknesses are significantly greater than in male than female children, and change with growth [34]. Therefore, in general, male children have certain advantages in tolerance to LASIK surgery. In our study, we confirmed that the threshold period of VDTt use is significantly higher in boys than in girls with a difference of 0.5 hours per day. Therefore, under the same conditions, girls’ daily use of VDT should be slightly lower than boys’, to better protect ocular surface health and prevent ocular surface diseases.

In addition, for boys, meibomian gland function was poorer in ABG than in NBG. Previous studies have shown that the meibomian gland function of subjects with high frequency incomplete blinking was significantly lower than that of normal subjects [35]. The TF instability associated with eyelid gland dysfunction provides an entry point for the vicious cycle of dry eye disease (DED), leading to hyperosmotic conditions and inflammatory responses on the ocular surface, which are both the cause and result of DED [36]. And the decrease of tear film thickness in dry eye patients after natural blinking was significantly faster than that in healthy subjects [37]. Therefore, this part of the population needed to constantly blink rapidly and compensatively increase the thickness of the tear film layer to prevent further water loss. Since one of the most important risk factors for abnormal blinking is the use of VDT, we infer that long-term use of VDT is also related to the function of the meibomian gland.

However, there are some limitations in our study. First, the indicator of abnormal blinking in this study is abnormal blinking frequency alone, without distinguishing other blinking ranges or forced blinking conditions. Second, the observation period of children's ocular surface morphology is relatively short, and longer observation time would help to compare the results with those of other studies and verify the present findings. Moreover, the sample size of each group in this study is small, and studies with larger samples will provide more certainty about the role of VDT use in abnormal blinking, and the relevant differences between genders. Finally, the children involved in this study were all from the eastern Chinese city of Shanghai, so it is difficult to exclude the effect of air pollution on ocular surface [38].

## Conclusions

All in all, the prolonged use of VDT is one of the most important risk factors for abnormal blinking in children. The cutoff values are 1.75 hours for boys and 1.25 hours for girls. Therefore, in clinical practice, if children have symptoms of abnormal blinking, ophthalmologists should carefully ask the children and their families about the use of VDT. Moreover, in daily life, more attention should be paid to appropriately reduce the use of VDT in children, especially girls. In addition, TF lipid layer is an important protective factor for children's use of VDT. Ophthalmologists should pay attention to the relationship between this index and children's use of VDT during clinical examination, timely check the stability of TF, so as to take appropriate intervention measures to reduce children's ocular surface injury.

## Data Availability

The datasets used and/or analyzed during the present study are available from the corresponding author on reasonable request.
